# Intervention, recruitment and evaluation challenges in the Bangladeshi community: Experience from a peer lead educational course

**DOI:** 10.1186/1471-2288-8-64

**Published:** 2008-10-09

**Authors:** SM Choudhury, S Brophy, MA Fareedi, B Zaman, P Ahmed, DRR Williams

**Affiliations:** 1 School of Medicine, Swansea University, Wales, UK; 2 Port-Tennant Surgery, Swansea, Wales, UK; 3 Peer educator, Swansea, Wales, UK

## Abstract

**Background:**

The incidence of Type 2 diabetes is increasing worldwide and diabetes is four times more common among ethnic minority groups than among the general Caucasian population. This study reflects on the specific issues of engaging people and evaluating interventions through written questionnaires within older ethnic minority groups.

**Methods:**

The original protocol set out to evaluate an adapted version of the X-PERT^® ^patient program  using questionnaires and interviews.

**Results:**

Questionnaires, even verbally completed, were unsuccessful and difficult to administer as participants found the questionnaire structure and design difficult to follow and did not perceive any benefit to completing the questionnaires. The benefits of attending the course were also poorly understood by participants and in many cases people participated in coming to the course as a favour to the researcher. Engaging participants required word of mouth and the involvement of active members of the community.

**Conclusion:**

Peer led courses and their evaluation in older ethnic minority communities needs a very different approach for that in younger Caucasian patients. A structured approached to evaluation (favoured by western educational system) is inappropriate. Engaging participants is difficult and the employment of local well known people is essential.

## Background

The prevalence of type 2 diabetes varies with ethnic origin. In the UK, people from the Indian subcontinent are particularly susceptible to diabetes [1-4] and one in five older South Asians have diabetes [5]. The adoption of self-management skills is necessary for people to manage their diabetes [6]. However, people from South Asian communities generally know less about their diabetes and its management compared to Caucasian patients [7]. A study of the British Bangladeshi diabetes population [8] found that informants expressed a strong desire to understand and explain the onset and experience of illness. Therefore, there is a real need for interventions targeted at ethnic minorities at high risk of diabetes. We undertook an evaluation of the X-PERT^® ^patient program  using questionnaires and interviews. However, we found out that there is also a need to understand the best methods of conducting an evaluation in older ethnic minority populations. The study reflects on the issue of using questionnaires to evaluate patient experiences and outcomes following an intervention.

## Methods

### Recruitment

Peer educators led a four hour educational session (based on the X-PERT^® ^patient programme [9]), exploring: what is diabetes; healthy eating and weight management; glycaemic index and possible complications of diabetes (with an emphasis on cardiovascular disease). The sessions were separate for men and women. The programme was evaluated by participant questionnaires, translated into Bangladeshi, and peer educator interviews. Participants also filled out a translated Summary of Diabetes Self-Care Activities Measure (SDSCA) questionnaire [10] at the start of the session and repeated a month after attendance at the course, to evaluate whether the participants changed any of their self-care activities after attending the educational session. Translations were performed by bilingual translators and back transcribed to ensure accuracy. The SDSCA questionnaire asks questions like "on how many days in the past 7 days did you eat high fat foods such as red meat or full-fat dairy produce". Cascade methodology was used (i.e. one person from each group becomes an educator and runs their own sessions). This method was chosen to reach as many people as possible while using a local person as the educator. Peer educators needed to: be Bangladeshi, have type 2 diabetes, be known in the community, have the time to run sessions, be enthusiastic and literate in English and Bangladeshi. The educational sessions were aimed at providing an overview of diabetes for better understanding and to empower participants in managing their diabetes. The primary aim of the study was to evaluate the course using written questionnaires and interviews. However, this paper reflects on the a posteriori experience of using written questionnaires with Bangladeshi people.

### Ethical approval

Ethical approval was obtained from the MREC for Wales.

## Results

There were 42 participants (67% female, average age 56 years and average disease duration 6.7 years). In the first session participants were not able to complete the questionnaires independently. Therefore, in the pilot it was found that written questionnaires could not be used. Instead, in the female sessions, the questionnaires were completed by volunteers and researchers sitting with the participant and discussing the questions and answers. Within the male sessions, questionnaires were completed in a 'class' setting with the peer educator reading out each question and discussing the range of answers. The main problems with the questionnaire were, many women were unable to read Bengali (or any written language), the questions themselves needed to be explained as they required a few conceptual steps (e.g. from the example from above, the understanding of what is a high fat food, the need to think of what was eaten (and did it fit the high fat food category) over seven days and add the days together) and many people did not understand why they were being asked to fill out this questionnaire they just wanted to get on with the course and to talk about diabetes. There was a general lack of understanding that diet and diabetes were linked ("diabetes is diabetes. It is an illness. Food is an eating thing..." Pt 2) and this meant the questionnaires were seen as irrelevant as well as complicated. Of 14 questionnaires sent to participants after the first two courses, 1 incomplete and 1 complete questionnaire were returned. Therefore, the methods were amended so that follow-up questionnaires were completed by visiting the participants in their home or telephoning participants and talking through the questions and interviewer recording the answers. At follow-up it was difficult to complete the questionnaires even interviewer assessed as the generic structure and purpose of questionnaires and forms was not well understood by participants. There was no concept of the relevance of evaluation in order to improve services and better meet the needs of others living with diabetes.

Engaging participants in attending the course and participating in the course evaluation was difficult. Many of the participants did not feel the complications of diabetes were a real issue to them personally (Session 3 Pt 2: "Why would people want to come to this class when they can just go to their doctors and get medication?") or that the management of the diabetes was their responsibility (Session 4 Pt 2 "It is a punishment from God for all the sins I've committed. What can I do now?"). People attending often came as a favour to the researchers (Session 1 Pt 1: "you are my daughters age, and if me attending this helps you, then it's good") or because the local Imam was the peer educator. No participants were recruited using posters, adverts in the Mosque or General Practitioner (GP) recommendations, although this did help in raising awareness about the study, especially the announcements in the Mosque. Much of the persuasion to encourage people to come to the educational course was through word of mouth.

## Discussion

This study is based on the posteriori experience of using the SDSCA questionnaire. The study did not set out as a primary objective to evaluate written questionnaires in the Bangladeshi community. However, findings suggest that the methods used to engage older people from ethnic minority groups needs to be different from those methods used for the Caucasian population. Using a questionnaire design is highly time consuming and off putting to participants. The manpower used in data collection through questionnaire could be better used in using qualitative methods which would be more acceptable to participants. For example, semi-structured interviews and focus or discussion groups were very well received by the participants of this study. A great deal of work is required to engage participants before any course of intervention programme is delivered. Many people in the older ethnic minority groups are likely to be in the pre-contemplation stage of behavioural change [11-13]. Much of the public health message regarding diabetes and obesity is delivered in English using the English media. Older ethnic minority patients are likely to miss these messages as they are more likely to use Satellite TV to watch Bangladeshi broadcasts, and read Bangladeshi newspapers. Recruitment to the course was solely using word of mouth. The choice of which local person is employed to advocate the course has a great impact on uptake rates. External researchers would find it difficult to run local courses in older ethnic minority communities. Therefore, peer educator courses using external patients would be unlikely to succeed in this population making 'roll out' of the course difficult.

Participants did not like completing questionnaires. This may have been because, with the educational section participants were receiving information, often new, in a language that they best understood and thus were benefiting them. However, with questionnaires, they were complicated and appeared irrelevant to participants in terms of a lack of understanding on the purpose of evaluation and on diet and diabetes. Further work to use quantitative methods would require developing questionnaires with Bangladesh people to ensure they are easy to understand and relevant to the prior knowledge and beliefs of people from a Bangladeshi community.

People from Bangladesh living in the UK usually are known to have family origins in the district of Sylhet and speak Sylheti, a dialect of Bangladeshi, which has no written form [14]. Sylheti Speakers often have difficulty understanding Bangladeshi as both differ quite significantly [15]. In previous research, written methods of data collection such as a community survey have been difficult to carry out, because of the known high illiteracy rates in both the English and Asian languages [16]. A lot of the participants attending the sessions were illiterate, especially amongst the women's group (in one session 4 out of seven attendees were illiterate in both English and Bangladeshi). Even interviewer delivery of the questionnaires which overcomes the literacy limitations was very challenging, as participants were able to hear each others personal details when forms were filled in for them by the researcher or peer educators. This posed a threat when trying to ensure confidentiality. Participants wanted the course delivered close to their homes, which limited the number of venues that could be used. This meant that private rooms could not be provided for separate interviews with each participant. This issue does highlight the importance of literacy in health care and the management of diabetes. Much of the information needs to be given in person, yet current health care information is largely written. However, solutions such as provision of I-Pods, DVDs or videos with information instead of information sheets perhaps could be investigated.

This study is based on the posteriori experience of using the SDSCA questionnaire. The study did not set out as a primary objective to evaluate written questionnaires in the Bangladeshi community. Therefore, the findings from this study report on observational evidence regarding one questionnaire.

## Conclusion

The use of written methods of evaluation may not be appropriate in older ethnic minority groups and interventions aimed at older ethnic minority groups should be designed with their specific needs in mind. Evaluation of interventions aimed at the Bangladeshi community need to be carefully planned and targeted to be relevant to this specific community. These findings may also be appropriate for other hard to reach communities.

## Abbreviations

GP: General practitioner; SDSCA questionnaire: Summary of Diabetes Self-Care Activities.

## Competing interests

The authors declare that they have no competing interests.

## Authors' contributions

All authors were involved in the design of the study. Data was collected by SC, BZ and PA. Analysis, interpretation and write up were initially undertaken by SC and SB and revisions and modifications were made by RW, MF, BZ and PA. All authors have given final approval for the submitted manuscript.

## Pre-publication history

The pre-publication history for this paper can be accessed here:


